# Modeling Metabolism and Disease in Bioarcheology

**DOI:** 10.1155/2015/548704

**Published:** 2015-08-06

**Authors:** Clifford Qualls, Otto Appenzeller

**Affiliations:** ^1^Health Sciences Center, University of New Mexico, Albuquerque, NM 87131, USA; ^2^New Mexico Health Enhancement and Marathon Clinics Research Foundation, Albuquerque, NM 87122, USA

## Abstract

We examine two important measures that can be made in bioarcheology on the remains of human and vertebrate animals. These remains consist of bone, teeth, or hair; each shows growth increments and each can be assayed for isotope ratios and other chemicals in equal intervals along the direction of growth. In each case, the central data is a time series of measurements. The first important measures are spectral estimates in spectral analyses and linear system analyses; we emphasize calculation of periodicities and growth rates as well as the comparison of power in bands. A low frequency band relates to the autonomic nervous system (ANS) control of metabolism and thus provides information about the life history of the individual of archeological interest. Turning to nonlinear system analysis, we discuss the calculation of SM Pinus' approximate entropy (ApEn) for short or moderate length time series. Like the concept that regular heart R-R interval data may indicate lack of health, low values of ApEn may indicate disrupted metabolism in individuals of archeological interest and even that a tipping point in deteriorating metabolism may have been reached just before death. This adds to the list of causes of death that can be determined from minimal data.

## 1. Introduction

Big data sets are revolutionizing science. They promote insights, facilitate comprehension, and order priorities for further studies using models and powerful computers. In the past decade important advances have been made using big data sets; they range from astronomy to climate change and from biology to geology. Bioarcheology, however, has not benefited from this trend, seemingly, because big data in bioarcheology are difficult to obtain.

Bioarcheology, as defined here, is cross-disciplinary research encompassing the study of human and animal remains. The best preserved tissues are bones, teeth, and occasionally hair.

Here we show that such archived materials provide sufficient data to model life's activities such as metabolism, growth, and biologic rhythms of individuals who have died decades or even millennia ago.

**Figure 1:**
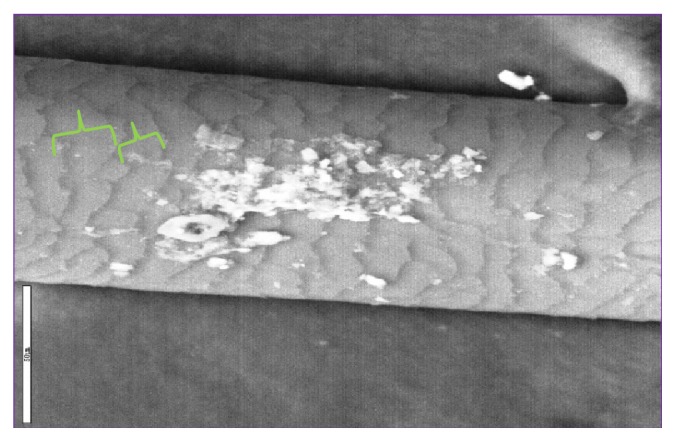
Human hair with repeat intervals (RIs) marked in green, 50 *μ*m vertical bar in white.

Many preserved tissues have growth marks left during life which reflect the rates of growth and by extension metabolism. For example, there are “scale like” markings on hair shafts which occur at more or less regular intervals which can be measured (Figure 1). Similarly on teeth surfaces or bone sections growth lines can easily be discerned. For all of these we use the term repeat intervals (RIs) from Bromage et al. [1] to denote the histological evidence on archived remains that betray life's activities such as metabolism and growth.

We hypothesized that the growth lines (GL) in hair, measured by microscopy as a time series, provide direct measurements of hair growth rate, which in turn depends on metabolism and therefore is a proxy for that individual's metabolism during life [1, 2]. By analogy heart rate time series variability provides insight into autonomic nervous system (ANS) function and can hint at diseased states [3, 4].

In death, forensic time series have been linked to ANS function and may reflect on the individual's life history; these time series include the repeat intervals between growth lines (RIs) in scalp hair expressed as sizes of hair scales measured by microscopy. Also the repeat intervals between* Perikymata Grooves *(PG) or* Striae of Retzius* (SR) in the enamel in human teeth and growth lines in archosaur teeth provide other time series [1, 2, 5]. In addition, there are time series of osteocyte density in bone [6]. Oxygen, hydrogen, or carbon isotope ratios as well as other chemicals in hair measured along fixed intervals in the direction of growth provide time series. Here we use spectral analysis of such time series as proxies of metabolism, which provide insight into dynamic processes in operation in the individual's past life.

## 2. Materials and Methods

The annual growth rate can often be computed in the time domain.

### 2.1. Annual Growth Rate and Preprocessing Forensic Time Series

The forensic time series may be discrete time *Y*_*i*_, *i* = 1,…, *N*, as in the growth lines in bone and teeth or for the scale sizes in hair, or a sample *Y*_*i*_ = *Y*(*t*_*i*_), *i* = 1,…, *N*, and *t*_*i*_ = *i*Δ*t* from a continuous time process such as chemicals measured in successive sections of bone of equal length Δ*t*. For the discrete time process take Δ*t* = 1, so that in both cases we have a discrete time series {*Y*_*i*_} of sample size (length) *N*. Usually this series will need to be preprocessed before it can be considered stationary Gaussian, the typical assumption for its spectral analysis.

Examining the plot *Y* versus time *t*, that is, *Y*_*i*_ versus *i*, it may show a nonzero mean, a trend over time, or an obvious annual cycle. We detrend the series if necessary by fitting a regression line ${\hat{Y}}_{i}=b+mt_{i}$ and replacing the series *Y*_*i*_ by its residuals $Y_{i}-{\hat{Y}}_{i}$ thereafter. The mean of the series is subtracted; the mean corresponds to the power at the zero frequency on the spectra, but our interest in spectral analysis sets aside consideration of the mean for separate analysis.

The next step in standardizing the time series {*Y*_*i*_} is to divide by its standard deviation. This preserves all the frequency content of the series and makes two different time series (perhaps even with different units of measurement) comparable. The situations where we would not standardize both series to variance = 1 is when our interest is the comparison of the variability (variances or the power in specified frequency bands) between the series.

If examination of the plot *Y* versus distance *t* along the hair shows an obvious annual cycle, then we can proceed directly to computing the annual growth rate of the hair.

**Example 1 (mammoth).**

The hydrogen isotope ratio measurements (*dD*) at multiples of 0.3 cm are taken along a hair from a mammoth [7, 8]. There is a partial annual sinusoid evident, whose periodicity is 52 weeks.

Fitting the annual sinusoid as well as a trend yields the function of length along the hair in cm: Predicted *dD* = −158 −0.727 ∗ cm + 8.69 ∗ sin (−0.196 ∗ cm + 3.98) as reported in [7]. The frequency of the sinusoid is 0.196 radians/cm. Converting radians to cycles we have frequency = (0.196 radians/cm)/(2*π* radians/cycle) = 0.0312 cycles/cm. This times the annual growth rate (cm/year) gives the number of cycles per year, which is equated to 1 cycle/year. Thus

(1) growth  rate=1(freq)(period)=1(0.0312 cycles/cm)(1 year/cycle⁡)=32 cm/yr.

This is the growth rate reported in Sharp et al. [7].

### 2.2. Computing Periodicities by Spectral Analysis of Forensic Time Series

To identified periodicities that are more frequent than annual and less frequent than daily we compute the power spectrum of the discrete time standardized version of our annually adjusted time series using SAS PROC SPECTRA and the Fast Fourier Transform [9]. The mean is removed because it corresponds only to the power at frequency = 0, which is not our interest. Dividing each series by its own standard deviation (SD) removes the last difference in units between series, which is appropriate if we are not interested in comparing variances. In other settings the comparison of means or variance may be the goal, so this information is retained for such an analysis. Note that *t*-axis is no longer measured in cm but in the number of Δ*t*, just as, in the mammoth Example 1 where Δ*t* = 0.3 cm. Now we give the spectral parameter definitions.

To be explicit, let the discrete time, stationary, Gaussian time series representing a series of measured intervals be {*Y*(*t*), for  *t* = 1, …, *N*} with continuous spectral density*f*(*λ*), where *λ* is the frequency on the *x*-axis. Then the periodogram *I*(*λ*) is an estimate of *f*(*λ*). One has

(2) $$\begin{array}{c} I\left(\lambda \right)=\frac{1}{2\pi N}{\left|\overset{N}{\underset{t=1}{\sum }}Y(t)e^{-i\lambda t}\right|}^{2},\;\text{for}\;\;-\pi <\lambda \leq \pi . \end{array}$$

Note that each sinusoid *e* ^*iλt*^ as a function of *t* has a frequency *λ* (radians per unit of *t*; in this case, radians per observation) and a corresponding period 2*π*/*λ*. Dividing *λ* by 2*π* radians per cycle gives a unit of cycle per observation as an alternative scale. For heartbeat, the frequency unit would be cycles per RR interval. For teeth, frequency units would be cycles per PG deposition (SR, Lines of Anderson (LA), or GL deposition). For the mammoth hair, the frequency units would be cycles per Δ*t* increment. The units of the periodogram (and the spectral density) can be seen from the fact (proof not shown) that the sum of *I*(*λ*_*j*_)Δ*λ*_*j*_ is the variance of the *Y*(*t*)'s. The unit for power density on the *y*-axis is the measurement unit squared divided by the unit of the *x*-axis.

There is a well-known problem with the periodogram as an estimator of the spectral density; it is not consistent; it does not become better as the sample size *N* gets larger. Thus, the usual (and better) estimate of *f*(*λ*) is the spectral density estimator $\hat{f}(\lambda )$, which is a smoothed and locally weighted average of the periodogram [10, 11]. One has

(3) f^λi=2N∑j=1N/2Wλi−λjIλj,for  λj=2πjN, j=1,…,N2.

The symbol [*x*] represents the integer part of *x*. Spectral density *f*(*λ*) is symmetric about *λ* = 0 by definition (definition not shown) and *I*(*λ*) is symmetric. Since *W*, called the spectral window, is taken to be symmetric, the estimated spectral density is symmetric, which allows one to plot the spectral density only for the nonnegative frequencies 0 ≤ *λ*_*j*_ ≤ 2*π*. Note that 4*π*/*N* = 2Δ*λ*_*j*_, where the extra 2 represents the sum over the negative *λ* and the *y*-axis should also be scaled by dividing by 2*π*, and finally that (4*π*/*N*) ÷ 2*π* = 2/*N* is the coefficient in (2).

**Figure 2:**
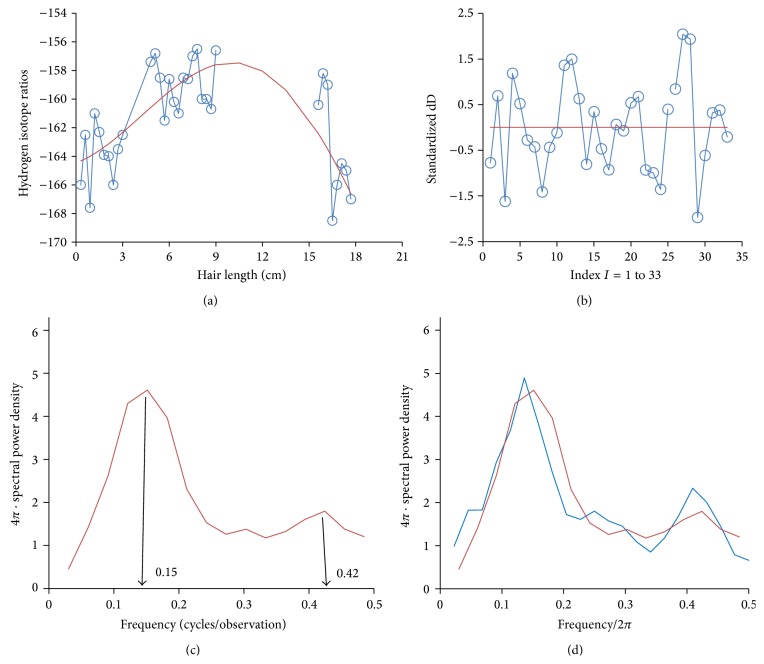
(a) Hydrogen isotope measured in 0.3 cm intervals of a hair of a Siberian mammoth loaned from the Smithsonian and published in [7]. (b) Standardized hydrogen isotope data in Figure 2(a) computed as the residuals of fitting the annual sinusoid, which have been standardized to mean = 0 and SD = 1. (c) Hydrogen isotope spectra of Smithonian mammoth (red line) hair measured every 0.3 cm. Series standardized to mean = 0 and SD = 1. The low frequency peak marked at 0.15 and high frequency peak at 0.42 are marked. Frequency axis (radians/observation) is divided by 2*π* radians/cycle to obtain cycles/observation. (d) Hydrogen isotope spectra of Smithonian mammoth (red line) and of Jarkov mammoth (blue line) hair measured every 0.3 cm. Series standardized to mean = 0 and SD = 1. Frequency axis is divided by 2*π*; multiplying *y*-axis by 2*π* maintains AUCs; multiplying *y*-axis by 2 represents contribution from negative frequencies.

Let us return to the mammoth example; the estimate of the spectral density of the standardized series in Figure 2(b) is Figure 2(c).

There are high frequency (0.42) and a low frequency (0.15) spectral peaks. Rearranging (1) above and including Δ*t* provide the formula for computing periodicity. One has

$$\begin{array}{cc} \left(1^{\prime }\right) & \text{period}=\frac{\Delta t}{\left(\text{freq}\right)\left(\text{growth}\;\;\text{rate}\right)}. \end{array}$$

For the low frequency peak, we compute a periodicity of 3.25 weeks. Consider

(4) Period=Δtfreqgrowth=0.3 cm/obs0.15 cycles/obs32 cm/52 wk=3.25 wk/cycle⁡,

where each observation represents one measurement interval with Δ*t* = 0.3 cm/obs.

Similarly, the periodicity of the high frequency peak is 1.2 weeks.

### 2.3. Nyquist Folding Frequency

There is a remaining issue; forensic time series are not measured continuously and the use of Δ*t* affects the computed spectral density; one cannot hope to measure frequencies higher than a certain value taking place within an interval of length Δ*t*. Furthermore, the spectral density is folded over at the Nyquist folding frequency *ω*_*N*_ with the high frequency content above *ω*_*N*_ being added to the low frequency content below *ω*_*N*_. For the Smithsonian mammoth hair,

(5) Nyquist  folding  frequencyωN=.5Δt=.5 cycles/obs0.3 cm/obs=1.67 cycles/cm.

This folding frequency times the growth rate gives a frequency of 1.03 cycles/week with a corresponding periodicity of 1/1.03 or approximately 1.0 week. Since we are not examining periodicities this low or lower, there may be no fold-back contamination in the above results. We have excluded the daily cycles from our interest; a much smaller Δ*t* would have been necessary for this purpose. Though we are not examining the daily cycles directly, it could be folded back and contaminate our spectral density computation. The high frequency that folds back to a low frequency is called an alias. The aliasing problem sometimes requires a detailed discussion. The aliases of a given frequency *λ* are *λ* + 2*kω*_*N*_, where *k* = ±1, ±2, ±3,…. The daily frequency is 7 cycles/week, and for *k* = −3, it is the alias of +0.82 cycles/week, but the observed peak is at 1/1.2 = 0.83 cycles/week. Now we are uncertain whether the observed high frequency peak is real at a periodicity 1.2 weeks or it is a contamination from the daily cycle at 1/7 weeks. See discussion. It is clear that one should formally consider the effect of the Nyquist folding frequency.

### 2.4. Low Frequency–High Frequency Ratio

To compute the power in a given band of frequencies, the spectral density is integrated over the band; that is, the spectral density times Δ*λ* is summed over the frequencies *λ*_*j*_ in the band.

Thus, the total power or power in frequency bands is obtained as areas under the curve where the units of the *x*-axis cancel. While the computation of power as areas (AUCs) under the spectral density from (2) above is typical, we also wish to compute the asymptotic standard error of such estimates. The formulae for power in a frequency band are adapted from Priestley [12, page 427]. One has

(6) AUC=∑λj  in  band2f^(λj)Δλj,Variance=∑λj  in  band4f^2(λj)Δλj2, where  Δλj=2πN,SE=Variance.

Again the factor of 2 represents the negative frequencies. When the time series is standardized, these formulae are dimensionless and can give a measure of the spectral shape. With these asymptotic means and SEs, one can compute a *t*-statistic and *P* value for the comparison of two AUCs. Note that these formulae in Priestley were developed for the periodogram *I*(*λ*) instead of the locally smoothed periodogram, the estimated spectral density function $\hat{f}(\lambda_{j})$. Both are approximately equal to the spectral density function *f*(*λ*). Koopmans [13] uses the estimated spectral densities $\hat{f}(\lambda_{j})$ for these formulae.

### 2.5. Distribution of Estimates

The distribution of the AUC estimator is based on the distribution of single estimated spectral densities $\hat{f}(\lambda_{j})$, which in turn depends on the effective degrees of freedom (EDF) of spectral window *W*(*λ*); see Koopmans [13, Table 8.1, page 279]. The standard result is $r(\hat{f}\left(\lambda \right)/f\left(\lambda \right))\approx \chi_{r}^{2}$, where the random variable on the left hand side is chi-square-distributed with *r* = EDF. Now we write the random AUC ≈ ∑_*λ*_*j*_  in  band_(2*f*(*λ*_*j*_)Δ*λ*_*j*_/*r*)*X*_*j*_, where *X*_*j*_ are independent *χ*_*r*_^2^, chi-square random variables. Since *EX*_*j*_ = *r*, the expected value of the random AUC is the targeted AUC. The variance of the random AUC is the targeted variance in (3). We now model the complicated distribution of the random AUC, a weighted sum of chi-square random variables, as a single distribution ≈ *cχ*_*R*_^2^ by the standard method of equating moments. One has estimates

(7) $$\begin{array}{c} E\left(c\chi_{R}^{2}\right)=cR,\;\;\text{Var}\left(c\chi_{R}^{2}\right)=2c^{2}R. \end{array}$$

In terms of the moments in (3) we have

(8) rf^(λ)f(λ)  is  approximately  distributed  as  a  χr2,chi-square  random  variable;AUC≈cχR2,  wherec=12Var(AUC)E(AUC)  and  R=2E2(AUC)Var(AUC)  withE(AUC),Var(AUC)  computed  as  themoments  of  AUC  in(3).

**Example 2 .**

Let us compute the 95% confidence intervals for the low frequency power for the Smithonian mammoth (red line, Figure 2(d)). First for the low frequency band, 0.07 ≤ *λ* < 0.27, we have AUC_2_ = 0.587, *V*_2_ = 0.064. Second, *c* = .5 ∗ .064/.587 = 0.0545, *R* = .587/.0545 = 10.8. Third, the upper and lower critical values for the *χ*_*R*_^2^ distribution are 3.7 and 21.6. Since *c*∗21.6 > 1, we compute the one-sided confidence interval with critical value 4.45. Finally, *P*[*c*∗4.45 < AUC] = 0.95 and [0.24, 1.0] is the 95% one-sided confidence interval for the power in this low frequency band. The upper bound is 1.0 since the area under the whole curve is 1.0 for the standardized series.

**Example 3 .**

Now the ratio (LF/HF) = AUC_2_/AUC_3_ = 0.587/0.322 = 1.82. Is this different than 1.0? Here, the high frequency band is 0.27 < *λ* < 0.5 with a width of 0.23, which introduces a bias relative to low frequency band width of 0.20. We could rerun our statistics for the comparable interval 0.30 < *λ* < 0.5, but we modify our estimate of AUC_3_ to be (0.20/0.23) ∗ 0.322 = 0.28 and LF/HF = 2.10. So, we develop the following two-tailed *F*-test. In spectral theory, the two AUCs are based on disjoint frequencies and are nearly independent. They would be more independent if the spectral windows did not overlap. We have already computed *c* = 0.0545 and *R* = 10.8 for the statistic AUC_2_.

For the modified AUC_3_, we have *E*(AUC_3_) = 0.28 and variance *V*_3_ = 0.0102. Second, *c*_3_ = 0.5 ∗ 0.0102/0.28 = 0.0182 and *R*_3_ = 0.28/0.0182 = 15.4. Third, the distribution of

(9) $$\begin{array}{c} \frac{\text{AU}{\text{C}}_{2}}{\text{AU}{\text{C}}_{3}}\approx \frac{c_{2}\chi_{R_{2}}}{c_{3}\chi_{R_{3}}}=\frac{c_{2}R_{2}}{c_{3}R_{3}}F_{R_{2},R_{3}},\\ \frac{c_{2}R_{2}}{c_{3}R_{3}}=\frac{0.0545*10.8}{0.0182*15.4}=2.10, \end{array}$$

where *F* = *F*_*R*_2_,*R*_3__ is an *F*-statistic. Finally, *P*[*F* > 2.1] = 0.09 and the two-sided *P* = 0.18.

For an example of comparison between two spectra, we add the data for a hair sample from a Jarkov Siberian mammoth (Figure 2(d), in blue). For the frequency band 0.07 ≤ *λ* < 0.27, including the low frequency peaks, we have AUC_2_ = 0.587 ± 0.253 (SE) for Smith and AUC_2_ = 0.527 ± 0.199 (SE) for Jarkov.

A test of the difference shows no difference:

(10) t=AUCSmith−AUCJarkovSESmith2+SEJarkov2=0.587−0.527.2532+.1992=0.060.322=0.19,  P=0.85.

*Comment*.  For a spectral analysis, these sample sizes are small: *N* = 33 and *N* = 44. For chemical analyses these can be larger.

### 2.6. Tipping Points and Telogen Duration (Quiescence in Growth)

Longer quiescence in hair growth (telogen [14]) indicates disrupted metabolism (longer intervals of oscillations of the system) and may be a marker of the tipping point in metabolism before complete cessations of rhythmic oscillations that are the hallmarks of biological systems. Here we compute the quiescent period in a basic model of reduced annual growth rate in hair. In normal human hair the telogen phase lasts approximately 3 months divided into approximately 4 periods. Hair grows for approximately 8 years and then, normally, falls out (metabolism ceases in this particular hair). Stress is known to lengthen the telogen; hormonal levels, age, and metabolism also affect the duration of the telogen. We cannot know at the time of modeling of the hair growth in what stage each hair is at the time of death. The basic model assumes (1) hair growth rate after the telogen continues independently of the telogen preceding it and independent of the telogen duration and (2) metabolism continues during the telogen phase as it was before and after, but since the hair is not growing, information about metabolism is missing in the hair record.

Let *Q* be the annual quiescence period measured in weeks and let *G* and *G*′ be the reference and reduced annual growth rates of the hair, respectively. *G*′ is reduced because of the augmented quiescence period *AQ*. The relationship is

(11) $$\begin{array}{c} G^{\prime }=\frac{52-AQ}{52-Q}G,\;\;\text{and}\;\;\text{conversely},\;\;AQ=52-\frac{G^{\prime }}{G}\left(52-Q\right). \end{array}$$

If *A* = 1 then *G*′ = *G* and *G*′ is not reduced; if *A* > 1 then *G*′ < *G* and *G*′ is reduced. For normal human hair growth, *Q* = 13 weeks. The augmented quiescence period *AQ* can only be computed by comparison of the reduced *G*′ to a reference *G* unless direct observation can be made in life.

**Example 4 .**

For the 16th century Spanish royals at the end of life, King Ferrante had an annual hair growth rate of 12 cm/year and Queen Isabella had a growth rate of 2 cm/year. Thus assuming *G* = 16 cm/year, the augmented quiescence periods were *AQ* = 52 − (12/16)(52 − 13) = 52 − 29.25 = 22.7 weeks for Ferrante and *AQ* = 52 − (2/16)(52 − 13) = 52 − 4.88 = 47.2 weeks for Isabella. These are longer than the normal 13 weeks and Isabella's is extreme. The historical record may contain information about metabolism; for example, Isabella had marked hair loss before she died. However, since direct measurement of quiescence was unlikely to be recorded, computations based on comparison of growth rates give information on quiescence periods as illustrated above.

**Figure 3:**
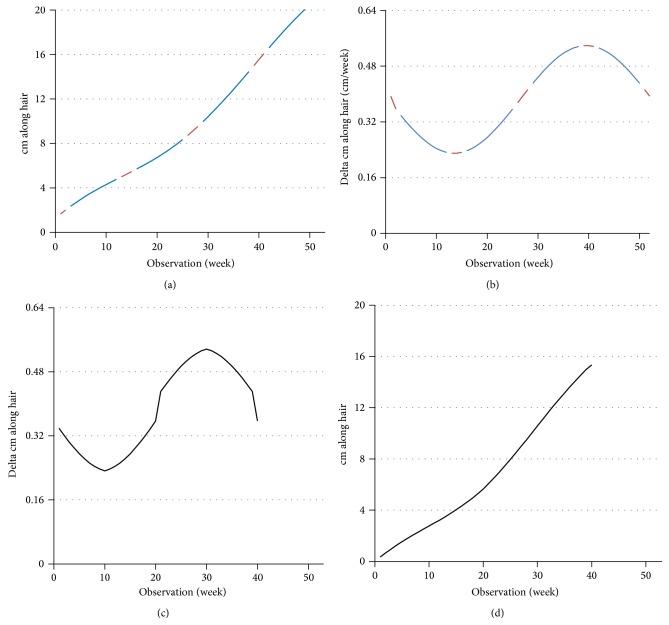
(a) Distance (cm) along a hypothetic hair (blue) that grows continuously for 52 weeks to a length of 20 cm as though there were no quiescence periods; now the quiescence periods are superimposed and marked (red). (b) Incremental weekly growth of the hypothetic hair (blue) with quiescence periods (red), mathematically obtained as the derivative of (a). (c) Observable incremental weekly growth for 39 weeks out of the year, periodic but not a sinusoid though a sinusoid of periodicity 39 weeks fits very well (not shown). (d) Distance (cm) along the observable hair for 39 weeks for an observed length of approximately 16 cm for the year, mathematically obtained as the integral of (c).

We now show graphically how the quiescence period *AQ* in our basic model affects the observed sinusoid representing the annual growth cycle. We will also check whether *AQ* affects the observed low frequency sinusoid representing autonomic neural system (ANS) control. Normal annual hair growth rate measured in hair growth is approximately 16 cm/year. Thus we begin with growth of 20 cm/year when there is no quiescence period (*Q* = 0 and *AQ* = 0) and weekly measurements that average 0.385 cm (see Figure 3(a)). The annual sinusoid as a function of weeks is followed by differentiation (see Figure 3(b)). Now three (3) months of quiescence are marked as missing (red) in Figures 3(a) and 3(b).

However the quiescence periods are not observed; thus the observable result is in Figures 3(c) and 3(d).

When the periodic function in Figure 3(c) is identified as an annual cycle, computations would consider the cycle as though on a 52-week *x*-axis. Thus, the annual growth rate is computed as 16 cm/year. We have shown how a growth rate of 20 cm/year becomes 16 cm/year in our basic model with *Q* = 3 months out of the year.

### 2.7. Nonlinear Time Series Analysis

We have examined spectral analysis in the frequency domain, which can be considered as linear systems analysis. There are also methods for nonlinear time series analyses and their application to chaos in dynamical systems [15]. The name most associated with this field is Takens [16]. The field provides measure of chaos, which can arise from nonlinear dynamical equations. There is also a more purely mathematical analysis [17]. We illustrate just one practical method from this large field.

#### 2.7.1. Approximate Entropy Measure

Approximate entropy (ApEn) as described by Pincus et al. [18, 19] quantifies regularity in time series data. ApEn and other measures have been used extensively in the analysis of biological time series [20]. In heart rate variability the low frequency/high frequency ratios reflect the autonomic nervous system (ANS) control of the activity of the cardiac pacemaker; in our analysis these ratios reflect the ANS pacemaker of metabolism and thus the ANS control of metabolism. In heart rate variability, disease such as diabetes decreases the variability; the heart rate is fixed at a higher rate but the variability in heart rate is reduced, a sign of ANS failure due to the disease. Conversely, high variability in ApEn reflects the robustness of the system. Bone, teeth, and hair also reflect metabolism and as such reflect ANS control.

ApEn depends on three parameters: the length of the time series (*N*), the width of the window that defines the patterns (*m*), and the tolerance that defines the closeness of the patterns (*r*). ApEn measures how the pattern (*m*) repeats itself within tolerance (*r*) over the course of the time series. ApEn(*m*, *r*) is a statistic that estimates the logarithmic difference for *m* and *m* + 1 in the conditional probability that runs of patterns that are close for previous repetitions remain close. Consequently, a large ApEn corresponds to an irregular time series and a small ApEn corresponds to a regular time series. ApEn for lengths of *N* > 50 has been found to be reproducible, but the literature suggests it can also be used with *N* ≤ 50. The pattern matching window can vary, but values between 1 and 3 are generally used. We used *m* = 2 and *m* + 1 = 3. A small tolerance value (*r*) corresponds to a fine pattern matching and a large *r* value corresponds to a coarser comparison. Our *r* was selected to be scale invariant as a percentage of the standard deviation of the time series being analyzed. We found values of *r* between 60% and 70% discriminated best for our analysis.

Thus, we use ApEn to measure the logarithmic likelihood that similar patterns of data length (*m*) that are similar remain so within a tolerance (*r*) on the next incremental (*m* + 1) comparison. In this analysis smaller values of ApEn indicate greater regularity in the data. Larger values are indicative of greater irregularities, more chaotic systems.

**Example 5 .**

The “Zweloo woman” was exhumed from a bog in Netherlands in 1951.

We examined six scalp hairs, 2000 years after her death. The approximate entropy (ApEn) was computed for the repeat intervals (RIs) defined by the sizes of hair scales along the length of the hair and for each of her six hairs separately; with *N* = 64–105 repeat intervals, pattern width *m* = 2 and tolerance *r* = 80% of the total standard deviation of the time series. The mean ApEn for Zweloo's hairs was 0.84 ± 0.05 (SD). Since tolerance (*r*) and, to a limited extent, pattern (*m*) are “free” parameters, these choices can be partially validated by comparison to a control group using the same parameters. Here a control group of 4 individuals had a mean ApEn of 0.71 ± 0.10.

## 3. Results and Discussion

For the methods outlined above, some operational aspects are now considered.

The Nyquist folding frequency probably is not a problem for measured RIs, since generally they are not sampling from a more continuous series. The RIs for hair are deposited in multiple of whole days with the multiple of days being related in an algometric fashion to the species' body mass; the whole days for periodic deposits to tooth enamel were 1 day for smallest bodied primates to 11 days for largest bodied primates and 8 days for humans [1]. Thus the daily cycles are not explicitly present in the RI data. This is not so for the continuous chemical record. The usual engineering solution to the Nyquist folding frequency problem is to design so that there is no power for frequencies beyond *ω*_*N*_. This is a consideration for the chemical times series, since the sampling rate Δ*t* may be under the experimenter's control. Even if this is not an option open to us, this spectral peak could still be real (uncontaminated), provided we knew the power of the daily cycle was low.

The data segments caused by missing values are pooled, laid end-to-end. The laying short time series end-to-end (concatenated) to form a longer series may cause difficulty. However, this difficulty is handled automatically in a similar situation for the spectral analysis of heart rate recordings where gaps occur due to technical problems or are introduced to eliminate periods of anomalous heartbeats for separate consideration. We follow this convention unless the difficulties become too large.

An important assumption for the distributions of spectral estimates in the section so named is that the choice of band width for the spectral window is wide enough for that the smoothed estimate of each spectral density function is consistent and narrow enough that estimates of adjacent spectral densities are approximately independent. The series length *N* must be large enough that the two conditions on the spectral window can be met.

Among several additional methods in use for nonlinear time series analysis, there are generalizations of the two basic methods (ApEn and FFT) used herein. First is the replacement of the deterministic rules used in approximate entropy (ApEn) with fuzzy logic rules yielding an improved algorithm (fApEn) that could help with the choice of the tolerance parameter (*r*) [21, 22]. Second is the Hilbert-Huang Transform (HHT) which is designed as a time-frequency analysis of nonlinear, nonstationary time series [23]. Compared to the spectral analysis using Fast Fourier Transform (FFT), which is designed as a frequency analysis of linear time series that are stationary in time, the HHT could help with periodicity estimation. The Hilbert-Huang Transform is implemented in a file exchange (hht) in MATLAB and in a package (hht) in the R language.

For our basic model of quiescence, we assume that the growth rate when the hair is growing is always constant and normal. Then the length of the quiescence intervals is the major effect on the annual growth rate and the effect is algebraic. If the disruption in metabolism affects both *Q* and the growth rate when the hair is growing, then we would need a model that connects increased quiescence to change (lowering) in the (instantaneous) growth rate when growing.

Does quiescence affect the frequency (periodicity) of the low frequency peak, the peak most related to autonomic nervous system (ANS) control? No doubt it does in the same manner that quiescence affects the annual growth cycle. Nonetheless, the computation of the periodicity of the low frequency peak is from the same hair growth record where we compute the growth rate usually reported. As such, it is comparable and useable on its face.
